# Chicago Medical Society

**Published:** 1866-05

**Authors:** 


### PROCEEDINGS OF MEDICAL SOCIETIES.

### CHICAGO MEDICAL SOCIETY—APRIL, 1866.

The fourteenth annual meeting of the Society was held on the evening of April 6th, 1866, at the residence of Dr. A. Groesbeck. The attendance was unusually large.

The following members were chosen officers for the ensuing year:

*President*—Dr. A. Groesbeck.
*Vice-President*—Dr. J. P. Ross.
*Secretary and Treasurer*—Dr. T. D. Fitch.

Delegates to the meeting of the American Medical Association, to be held at Baltimore, May 1, 1866, were chosen as follows: Drs. Chas. G. Smith, R. C. Hamill, T. D. Fitch, N. S. Davis, and W. D. Faxon.

The following were appointed delegates to the meeting of the

Illinois State Medical Society, to be held at Decatur, Tuesday, June 5th: Drs. E. L. Holmes, J. P. Ross, S. Wickersham, R. C. Hamill, S. C. Blake, G. C. Paoli, T. J. Bluthardt, S. J. Avery, D. B. Trimble, N. T. Quales, Ira Hatch, W. L. Faxon, and A. Fisher.

After the transaction of business, the members were invited by the host to partake of the bounties of the supper table, richly loaded with all the delicacies the heart (!) could wish.

During the past year the Society has enjoyed more than ordinary prosperity. The meetings have been generally well attended, and the discussions interesting and profitable. The papers which have been read from time to time, the reports of cases, and the pathological specimens exhibited by different members, would do credit to the medical society of any city.

Few physicians in Chicago can well afford to lose the benefit which directly and indirectly the attendance upon the meetings of this Society ensures.

We trust efforts will be made during the coming year to render the meetings of the Society even more instructive than they have been. This can be done if members will present the fruits of their experience, whether derived from private practice or from the different hospitals, infirmaries or dispensaries of the city.

At the meeting of April 13th, Drs. R. G. Bogue and B. Durham, Jr., were elected members of the Society.

Dr. Davis gave a verbal report, regarding the sanitary condition of the city during the past two months. There had been nothing peculiar in the weather to induce an unusual prevalence of disease, although catarrhal affections had been common. He had not observed any tendency to affections of the bowels, which could be considered as precursory to cholera. The city must be regarded as unusually healthy. The deaths during the past month were less in proportion to the population, than during the corresponding season for the past ten years; and yet the condition of our city was disgraceful in the extreme. Although there was an unpardonable neglect in removing the unusual amount of filth from the streets, alleys and yards, the temperature had been so low that these accumulations were now comparatively harmless. Almost every vacant lot was covered with water, which is also found stagnant under nearly two-thirds of all the dwellings of the city. This, also, was comparatively innocuous during cold weather; but as the temperature becomes elevated and the ground partially dried, the emanations from the filth everywhere found in the city could not but prove prejudicial to the health of our populace. Attention was called to the importance of removing all vegetable and animal substances scattered so profusely around dwellings, as, also, of draining yards and vacant lots in such a way that the surface water may be rapidly conducted into the sewers.

Dr. J. Bartlett read an interesting report on cholera with statistics.

### CHOROIDAL TUMOR.

Dr. Holmes exhibited a tumor of the choroid, with the following history: Two years ago a lady, about 50 years of age, consulted him about a pain in and near the right eye, which had already existed some weeks. Vision was totally destroyed; the pupil contracted and filled with exudation; the iris discolored and lying almost in contact with the cornea which was still transparent; the globe was abnormally hard. Extirpation of the eye was advised, to which, however, the patient was unwilling to submit. For two years, she was under the treatment of various practitioners, who endeavored in vain to relieve her suffering. Her health had become much impaired. On recently applying to Dr. H. again, she was willing to submit to the operation of extirpation. The globe divided through its centre, was found to be filled with solid material. The structure of the tumor was principally fibroid cells and exudation corpuscles, which could be seen in the specimen under the microscope kindly prepared by Dr. Lyman.

Such tumors are the products of hypoplastic choroiditis, and are non-malignant in character. After the removal of the eye, the patient at once recovered her health.

### ELEPHANTIASIS OF THE CLITORIS.

Dr. Lyman exhibited a specimen of this rare disease, which was removed by Dr. McClure, from a patient at the Alms

House. She was a woman about twenty-five years of age, the mother of several children, and had suffered with constitutional syphilis. The clitoris commenced to enlarge about three years previous to the time of its removal. It formed a pendulous bifid tumor, dangling between the thighs, and reaching half way to the knees. Its surface was nodulated, and was ulcerated at several points, exuding a most offensive discharge. The tumor was easily removed by amputation after the application of a whip-cord ligature around its pedicle. The stump cicatrized in eight days after the operation. Microscopical examination showed fibrous and connective tissue as the principal elements of the mass.

Dr. Hildreth described the peculiar effects of ℥vi. of chemically pure sulphuric ether administered to a patient at the Cook County Hospital. Perfect anæsthesia was produced in ten minutes. The patient appeared as if under the influence of a poisonous dose of opium, the muscles being perfectly relaxed, pulse 80. He was unconscious, and unable to swallow fluids placed in the mouth. Coffee and brandy introduced into the rectum, and flagellation, with strong aqua ammonia held to the nostrils, aroused him. In three and a half hours the patient had fully recovered.

### FRACTURE OF THE BASE OF THE SKULL.

Dr. McAllister related the history of a case in which a gentleman had been thrown from a carriage, striking his head against a railroad tie. On visiting him a few hours after the accident, he found him insensible, with blood flowing from the right ear. The discharge soon became serous in character. In sixty hours, consciousness returned; in seven days the patient was apparently in perfect health, and able to attend to his ordinary business.

Several members in reference to this case, expressed an opinion that there was undoubtedly a fracture of the base of the skull, and that the patient was not yet out of danger.

Dr. J. W. Brooks stated that one of his own children speedily recovered from a severe injury of the head, accompanied by bleeding from the ears and nose. The child appeared perfectly well, and was able to attend school. Death occurred, however, five months after the accident. An examination revealed an abscess of the brain, with fracture of the base of the skull.

Dr. Tucker stated that he had sometime since treated a child, who had fallen into the hold of a vessel, producing a discharge of blood from the nose and ears. The recovery was speedy, and the health was good years after the injury.

Allusion was made to the fallacy in diagnosis in these cases, if the blood from the nose should flow into the ears, as could readily happen in certain positions of the head.

### TRICHINA SPIRALIS.

Dr. Lyman exhibited under the microscope, specimens of trichina spiralis, taken from pork cured at the packing houses of Chicago. Of 1394 swine examined, trichinæ were found in twenty-eight.

### SYPHILIS.

Dr. C. G. Smith related the particulars of a case of syphilis communicated by an infant to its wet nurse. When the nurse took charge of the child, it had eruptions on the surface, and a sore mouth, of the character of which, however, she was ignorant. In three weeks she discovered indurated ulcers around the nipples. In two months there appeared a syphilitic roseola on the neck and arms. Mercurial inunctions for ten days, caused the eruptions to disappear, and the ulcers to heal, although the indurations remained.

A member related the history of a young woman, whose irreproachable character left no doubt of the truth of her narrative, who experienced the horrors of syphilitic innoculation, through a kiss from the gentleman to whom she was engaged. A chancre upon the lip was the result of this caress, and subsequent medical investigation revealed the fact that the young man was at the time under treatment for syphilitic ulceration of the throat.

At the meeting of April 27th, Drs. D. T. Nelson, Wm. Knox, Geo. K. Amerman and E. Ingals, were elected members of the society.

